# [2,6-Bis(5-eth­oxy-1,3-oxazol-2-yl)-4-meth­oxy­phenyl-κ^3^ *N*,*C* ^1^,*N*′]bromidopalladium(II)

**DOI:** 10.1107/S1600536812049197

**Published:** 2012-12-08

**Authors:** Wen-Hui Nan, Jian-Ping Tan, Qun-Li Luo

**Affiliations:** a Key Laboratory of Applied Chemistry of Chongqing Municipality, College of Chemistry and Chemical Engineering, Southwest University, Chongqing 400715, People’s Republic of China

## Abstract

In the title compound, [PdBr(C_17_H_17_N_2_O_5_)], the Pd^II^ atom is coordinated by an *N*,*C* ^1^,*N*′-tridentate pincer ligand and a Br atom in a distorted square-planar geometry. In the crystal, mol­ecules are connected by C—H⋯Br and C—H⋯O hydrogen bonds, and π–π inter­actions between the oxazole and benzene rings [centroid–centroid distance = 3.7344 (19) Å], resulting in a three-dimensional supra­molecular structure.

## Related literature

For background to pincer palladium complexes, see: van Koten & Gebbink (2011 ▶); Moreno *et al.* (2010 ▶); Selander & Szabó (2011 ▶). For palladium complexes with NCN pincer ligands, see: Hao *et al.* (2010 ▶); Young *et al.* (2011 ▶). For studies on the chemistry of bis­(oxazole) pincer palladium complexes, see: Luo *et al.* (2007 ▶, 2011 ▶); Xu *et al.* (2011 ▶). For structures of related bis­(azole) pincer palladium complexes, see: Ghorai *et al.* (2012 ▶); Luo *et al.* (2012 ▶).
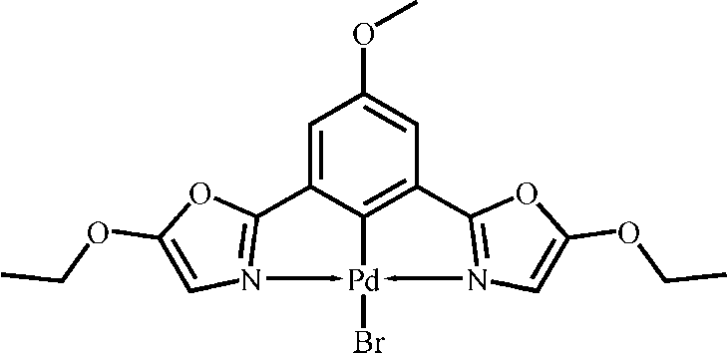


## Experimental

### 

#### Crystal data

[PdBr(C_17_H_17_N_2_O_5_)]
*M* *_r_* = 515.64
Triclinic, 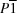

*a* = 9.0209 (3) Å
*b* = 9.6544 (3) Å
*c* = 10.9200 (3) Å
α = 87.093 (2)°
β = 86.974 (1)°
γ = 85.793 (2)°
*V* = 946.11 (5) Å^3^
*Z* = 2
Mo *K*α radiation
μ = 3.12 mm^−1^
*T* = 296 K
0.43 × 0.41 × 0.37 mm

#### Data collection

Bruker APEX CCD diffractometer
Absorption correction: multi-scan (*SADABS*; Sheldrick, 1996 ▶) *T*_min_ = 0.347, *T*_max_ = 0.391
15776 measured reflections
4311 independent reflections
3770 reflections with *I* > 2σ(*I*)
*R*_int_ = 0.034

#### Refinement

*R*[*F* ^2^ > 2σ(*F* ^2^)] = 0.036
*wR*(*F* ^2^) = 0.113
*S* = 1.08
4311 reflections
235 parameters
6 restraints
H-atom parameters constrained
Δρ_max_ = 0.72 e Å^−3^
Δρ_min_ = −1.25 e Å^−3^

### 

Data collection: *SMART* (Bruker, 2007 ▶); cell refinement: *SAINT* (Bruker, 2007 ▶); data reduction: *SAINT*; program(s) used to solve structure: *SHELXS97* (Sheldrick, 2008 ▶); program(s) used to refine structure: *SHELXL97* (Sheldrick, 2008 ▶); molecular graphics: *XP* in *SHELXTL* (Sheldrick, 2008 ▶); software used to prepare material for publication: *SHELXTL*.

## Supplementary Material

Crystal structure: contains datablock(s) I, global. DOI: 10.1107/S1600536812049197/hy2606sup1.cif

Structure factors: contains datablock(s) I. DOI: 10.1107/S1600536812049197/hy2606Isup2.hkl

Additional supplementary materials:  crystallographic information; 3D view; checkCIF report

## Figures and Tables

**Table 1:** Selected bond lengths (Å)

Pd1—C11	1.954 (3)
Pd1—N1	2.056 (3)
Pd1—N2	2.055 (3)
Pd1—Br1	2.4941 (4)

**Table 2:** Hydrogen-bond geometry (Å, °)

*D*—H⋯*A*	*D*—H	H⋯*A*	*D*⋯*A*	*D*—H⋯*A*
C2—H2*A*⋯Br1^i^	0.97	2.80	3.526 (5)	132
C16—H16*A*⋯O2^ii^	0.96	2.44	3.391 (5)	170

Symmetry codes: (i) 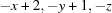; (ii) 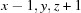.

**Fig. 1.**
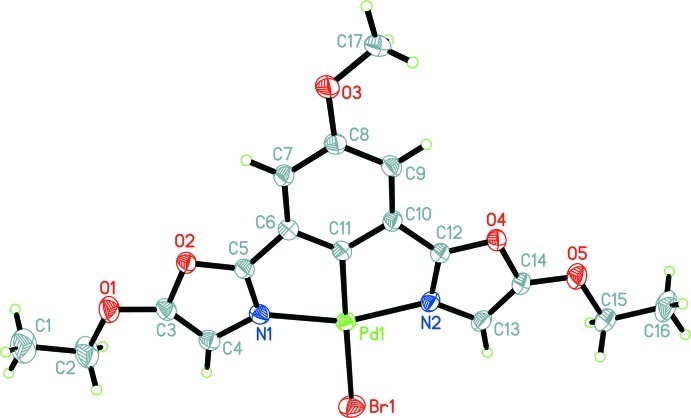
The molecular structure of the title compound. Displacement ellipsoids are drawn at the 30% probability level.

## supplementary crystallographic information

### Comment

Cross-coupling reactions catalyzed by palladium complexes are among the most important tools for C—C bond construction. Considerable attention has recently been devoted to pincer-Pd complexes due to their catalytic abilities (Ghorai *et al.*, 2012; van Koten & Gebbink, 2011; Moreno *et al.*, 2010; Selander & Szabó, 2011). We are interested in the NCN type of pincer-Pd complexes (Luo *et al.*, 2007, 2011, 2012; Xu *et al.*, 2011) as a variety of nonphosphine pincer catalytic system, that contains two nitrogen atoms as donating sites in the coordination sphere (Hao *et al.*, 2010; Young *et al.*, 2011)

The title compound was conveniently synthesized from the reaction of Pd(dba)_2_ (dba = dibenzylideneacetone) with 1-bromo-2,6-bis(5-ethoxyoxazol-2-yl)-4-methoxy benzene in dry benzene under reflux in an argon atmosphere. As a result, the title compound was isolated with 84% yield. Suitable single crystals were grown *via* vapor diffusion of hexane into a DMF solution of the soluble reaction product at room temperature for dozens of days.

The molecular structure is shown in Fig. 1 and selected bond lengths in Table 1. In the crystal, the molecules are linked by intermolecular C—H···Br and C—H···O hydrogen bonds (Table 2) and π–π interactions between the oxazole and benzene rings [centroid–centroid distance = 3.7344 (19) Å], resulting in a three-dimensional supramolecular structure.

### Experimental

Under an argon atmosphere, a 25 ml Schlenk flask was charged with 1-bromo-2,6-bis(5-ethoxyoxazol-2-yl)-4-methoxybenzene (106 mg, 0.3 mmol), Pd(dba)_2_ (173 mg, 0.3 mmol) and dry benzene (15 ml). The reaction mixture was heated and refluxed for 2 h, and then cooled to room temperature and stirred for further 2 h. The resultant mixture was directly transferred on to a diatomite column and eluted first with hexane to remove dibenzylideneacetone and then with chloroform. The collected target compound was crystallized from CHCl_3_/MeOH as a slight yellow solid (yield: 84%). ^1^H NMR (300 MHz, CDCl_3_): δ 6.76 (s, 2H), 6.52 (s, 2H), 4.25 (q, 4H, *^3^J* = 6.9 Hz), 3.83 (s, 3H), 1.49 (t, 6H, *^3^J* = 7.0 Hz). ^13^C NMR (75 MHz, CDCl_3_): δ159.3, 158.5, 157.6, 154.7, 129.9, 107.3, 100.3, 55.8, 14.4. LRMS (ESI): *m/z*(%) 951 (100) (2*M*^+^–Br).

### Refinement

H atoms were positioned geometrically and refined as riding atoms, with C–H = 0.93 (aromatic), 0.96 (CH_3_) and 0.97 (CH_2_) Å and with *U*_iso_(H) = 1.2(1.5 for methyl)*U*_eq_(C).

### Crystal data

**Table:**

[PdBr(C_17_H_17_N_2_O_5_)]	*Z* = 2
*M**_r_* = 515.64	*F*(000) = 508
Triclinic, *P*1	*D*_x_ = 1.810 Mg m^−^^3^
Hall symbol: -P 1	Mo *K*α radiation, λ = 0.71073 Å
*a* = 9.0209 (3) Å	Cell parameters from 15776 reflections
*b* = 9.6544 (3) Å	θ = 1.9–27.5°
*c* = 10.9200 (3) Å	µ = 3.12 mm^−^^1^
α = 87.093 (2)°	*T* = 296 K
β = 86.974 (1)°	Block, yellow
γ = 85.793 (2)°	0.43 × 0.41 × 0.37 mm
*V* = 946.11 (5) Å^3^	

### Data collection

**Table:**

Bruker APEX CCD diffractometer	4311 independent reflections
Radiation source: fine-focus sealed tube	3770 reflections with *I* > 2σ(*I*)
Graphite monochromator	*R*_int_ = 0.034
Detector resolution: 0.01 pixels mm^-1^	θ_max_ = 27.5°, θ_min_ = 1.9°
φ and ω scans	*h* = −11→11
Absorption correction: multi-scan (*SADABS*; Sheldrick, 1996)	*k* = −12→12
*T*_min_ = 0.347, *T*_max_ = 0.391	*l* = −14→13
15776 measured reflections	

### Refinement

**Table:**

Refinement on *F*^2^	Primary atom site location: structure-invariant direct methods
Least-squares matrix: full	Secondary atom site location: difference Fourier map
*R*[*F*^2^ > 2σ(*F*^2^)] = 0.036	Hydrogen site location: inferred from neighbouring sites
*wR*(*F*^2^) = 0.113	H-atom parameters constrained
*S* = 1.08	*w* = 1/[σ^2^(*F*_o_^2^) + (0.080*P*)^2^] where *P* = (*F*_o_^2^ + 2*F*_c_^2^)/3
4311 reflections	(Δ/σ)_max_ < 0.001
235 parameters	Δρ_max_ = 0.72 e Å^−^^3^
6 restraints	Δρ_min_ = −1.25 e Å^−^^3^

### Special details

**Table:**

Geometry. All e.s.d.'s (except the e.s.d. in the dihedral angle between two l.s. planes) are estimated using the full covariance matrix. The cell e.s.d.'s are taken into account individually in the estimation of e.s.d.'s in distances, angles and torsion angles; correlations between e.s.d.'s in cell parameters are only used when they are defined by crystal symmetry. An approximate (isotropic) treatment of cell e.s.d.'s is used for estimating e.s.d.'s involving l.s. planes.
Refinement. Refinement of *F*^2^ against ALL reflections. The weighted *R*-factor *wR* and goodness of fit *S* are based on *F*^2^, conventional *R*-factors *R* are based on *F*, with *F* set to zero for negative *F*^2^. The threshold expression of *F*^2^ > σ(*F*^2^) is used only for calculating *R*-factors(gt) *etc*. and is not relevant to the choice of reflections for refinement. *R*-factors based on *F*^2^ are statistically about twice as large as those based on *F*, and *R*- factors based on ALL data will be even larger.

### Fractional atomic coordinates and isotropic or equivalent isotropic displacement parameters (Å^2^)

**Table:**

	*x*	*y*	*z*	*U*_iso_*/*U*_eq_	
Pd1	0.75135 (2)	0.11221 (2)	0.04359 (2)	0.03536 (11)	
Br1	0.73062 (5)	0.33747 (4)	0.14750 (4)	0.06338 (15)	
O1	1.1647 (3)	0.2444 (3)	−0.3330 (3)	0.0641 (8)	
O2	1.0326 (3)	0.0693 (2)	−0.2594 (2)	0.0427 (5)	
O3	0.7890 (3)	−0.4275 (2)	−0.2212 (3)	0.0579 (7)	
O4	0.4921 (3)	−0.1921 (2)	0.1850 (2)	0.0442 (5)	
O5	0.3381 (3)	−0.1541 (3)	0.3498 (2)	0.0668 (8)	
N1	0.9050 (3)	0.1515 (3)	−0.0974 (2)	0.0396 (6)	
N2	0.6019 (3)	0.0043 (3)	0.1522 (2)	0.0400 (6)	
C1	1.3044 (8)	0.4175 (6)	−0.4370 (6)	0.106 (2)	
H1A	1.3264	0.5134	−0.4395	0.158*	
H1B	1.2634	0.3971	−0.5128	0.158*	
H1C	1.3941	0.3595	−0.4253	0.158*	
C2	1.1945 (6)	0.3901 (4)	−0.3332 (5)	0.0810 (14)	
H2A	1.2346	0.4105	−0.2560	0.097*	
H2B	1.1033	0.4483	−0.3439	0.097*	
C3	1.0668 (4)	0.2043 (3)	−0.2459 (3)	0.0447 (7)	
C4	0.9916 (4)	0.2562 (3)	−0.1487 (3)	0.0426 (7)	
H4A	0.9957	0.3450	−0.1203	0.051*	
C5	0.9345 (3)	0.0438 (3)	−0.1655 (3)	0.0372 (6)	
C6	0.8596 (3)	−0.0824 (3)	−0.1380 (3)	0.0372 (6)	
C7	0.8675 (4)	−0.2063 (3)	−0.1977 (3)	0.0414 (7)	
H7A	0.9331	−0.2201	−0.2653	0.050*	
C8	0.7764 (4)	−0.3086 (3)	−0.1552 (3)	0.0436 (7)	
C9	0.6796 (4)	−0.2931 (3)	−0.0517 (3)	0.0412 (7)	
H9A	0.6196	−0.3635	−0.0233	0.049*	
C10	0.6764 (3)	−0.1684 (3)	0.0073 (3)	0.0376 (7)	
C11	0.7643 (3)	−0.0646 (3)	−0.0372 (3)	0.0358 (6)	
C12	0.5894 (3)	−0.1220 (3)	0.1135 (3)	0.0384 (6)	
C13	0.5062 (4)	0.0193 (4)	0.2543 (3)	0.0442 (7)	
H13A	0.4905	0.0973	0.3011	0.053*	
C14	0.4399 (4)	−0.1014 (4)	0.2730 (3)	0.0464 (8)	
C15	0.2843 (5)	−0.0696 (5)	0.4494 (4)	0.0643 (11)	
H15A	0.2321	0.0153	0.4183	0.077*	
H15B	0.3666	−0.0450	0.4957	0.077*	
C16	0.1823 (6)	−0.1520 (5)	0.5285 (4)	0.0837 (16)	
H16A	0.1440	−0.0984	0.5963	0.126*	
H16B	0.2351	−0.2356	0.5588	0.126*	
H16C	0.1013	−0.1755	0.4817	0.126*	
C17	0.6779 (5)	−0.5245 (4)	−0.1992 (4)	0.0587 (10)	
H17A	0.6999	−0.6018	−0.2508	0.088*	
H17B	0.5824	−0.4801	−0.2174	0.088*	
H17C	0.6766	−0.5571	−0.1147	0.088*	

### Atomic displacement parameters (Å^2^)

**Table:**

	*U*^11^	*U*^22^	*U*^33^	*U*^12^	*U*^13^	*U*^23^
Pd1	0.03735 (16)	0.03482 (16)	0.03421 (16)	−0.00236 (10)	−0.00149 (10)	−0.00492 (10)
Br1	0.0765 (3)	0.0515 (2)	0.0634 (3)	−0.0026 (2)	−0.0009 (2)	−0.0195 (2)
O1	0.0782 (19)	0.0491 (15)	0.0652 (17)	−0.0235 (14)	0.0259 (15)	−0.0074 (13)
O2	0.0477 (12)	0.0354 (11)	0.0450 (12)	−0.0084 (9)	0.0083 (10)	−0.0061 (9)
O3	0.0664 (16)	0.0390 (13)	0.0692 (17)	−0.0145 (11)	0.0214 (13)	−0.0205 (12)
O4	0.0478 (12)	0.0430 (12)	0.0422 (12)	−0.0101 (10)	0.0083 (10)	−0.0052 (10)
O5	0.085 (2)	0.0609 (17)	0.0537 (16)	−0.0198 (15)	0.0327 (14)	−0.0159 (13)
N1	0.0395 (14)	0.0366 (14)	0.0430 (15)	−0.0045 (11)	−0.0020 (11)	−0.0040 (11)
N2	0.0401 (14)	0.0455 (15)	0.0341 (13)	−0.0022 (11)	0.0030 (11)	−0.0050 (11)
C1	0.137 (5)	0.074 (3)	0.105 (4)	−0.041 (3)	0.041 (4)	0.002 (3)
C2	0.099 (4)	0.046 (2)	0.097 (4)	−0.021 (2)	0.031 (3)	0.002 (2)
C3	0.0479 (17)	0.0368 (16)	0.0498 (19)	−0.0109 (13)	0.0022 (15)	0.0012 (14)
C4	0.0460 (17)	0.0358 (16)	0.0465 (18)	−0.0063 (13)	−0.0014 (14)	−0.0026 (13)
C5	0.0371 (15)	0.0365 (15)	0.0382 (16)	−0.0038 (12)	−0.0014 (12)	−0.0021 (12)
C6	0.0366 (15)	0.0359 (15)	0.0391 (16)	−0.0016 (12)	−0.0028 (13)	−0.0008 (12)
C7	0.0407 (16)	0.0411 (17)	0.0419 (17)	−0.0036 (13)	0.0063 (13)	−0.0057 (13)
C8	0.0475 (18)	0.0355 (16)	0.0480 (19)	−0.0047 (13)	0.0041 (15)	−0.0090 (14)
C9	0.0419 (16)	0.0346 (15)	0.0471 (18)	−0.0078 (13)	0.0031 (14)	−0.0017 (13)
C10	0.0364 (15)	0.0415 (16)	0.0346 (16)	−0.0018 (12)	0.0016 (12)	−0.0029 (13)
C11	0.0336 (14)	0.0361 (15)	0.0380 (16)	−0.0044 (11)	−0.0021 (12)	−0.0007 (12)
C12	0.0405 (16)	0.0390 (16)	0.0359 (16)	−0.0048 (12)	0.0022 (13)	−0.0042 (12)
C13	0.0464 (18)	0.053 (2)	0.0333 (16)	−0.0022 (15)	0.0019 (14)	−0.0073 (14)
C14	0.0500 (19)	0.0526 (19)	0.0363 (17)	−0.0082 (15)	0.0096 (14)	−0.0059 (14)
C15	0.075 (3)	0.076 (3)	0.044 (2)	−0.017 (2)	0.0166 (19)	−0.0172 (18)
C16	0.112 (4)	0.081 (3)	0.054 (3)	−0.005 (3)	0.040 (3)	−0.011 (2)
C17	0.067 (2)	0.047 (2)	0.064 (2)	−0.0161 (18)	0.003 (2)	−0.0165 (18)

### Geometric parameters (Å, º)

**Table:**

Pd1—C11	1.954 (3)	C3—C4	1.328 (5)
Pd1—N1	2.056 (3)	C4—H4A	0.9300
Pd1—N2	2.055 (3)	C5—C6	1.447 (4)
Pd1—Br1	2.4941 (4)	C6—C11	1.371 (5)
O1—C3	1.326 (4)	C6—C7	1.387 (4)
O1—C2	1.451 (5)	C7—C8	1.378 (4)
O2—C5	1.344 (4)	C7—H7A	0.9300
O2—C3	1.378 (4)	C8—C9	1.399 (5)
O3—C8	1.380 (4)	C9—C10	1.392 (5)
O3—C17	1.425 (4)	C9—H9A	0.9300
O4—C12	1.342 (4)	C10—C11	1.377 (4)
O4—C14	1.374 (4)	C10—C12	1.438 (4)
O5—C14	1.323 (4)	C13—C14	1.350 (5)
O5—C15	1.435 (5)	C13—H13A	0.9300
N1—C5	1.312 (4)	C15—C16	1.475 (6)
N1—C4	1.400 (4)	C15—H15A	0.9700
N2—C12	1.325 (4)	C15—H15B	0.9700
N2—C13	1.380 (4)	C16—H16A	0.9600
C1—C2	1.492 (7)	C16—H16B	0.9600
C1—H1A	0.9600	C16—H16C	0.9600
C1—H1B	0.9600	C17—H17A	0.9600
C1—H1C	0.9600	C17—H17B	0.9600
C2—H2A	0.9700	C17—H17C	0.9600
C2—H2B	0.9700		
			
C11—Pd1—N2	79.26 (12)	C8—C7—H7A	120.6
C11—Pd1—N1	79.31 (11)	C6—C7—H7A	120.6
N2—Pd1—N1	158.57 (11)	C7—C8—O3	115.2 (3)
C11—Pd1—Br1	179.10 (9)	C7—C8—C9	122.1 (3)
N2—Pd1—Br1	100.00 (8)	O3—C8—C9	122.7 (3)
N1—Pd1—Br1	101.42 (7)	C10—C9—C8	117.5 (3)
C3—O1—C2	114.6 (3)	C10—C9—H9A	121.3
C5—O2—C3	104.3 (2)	C8—C9—H9A	121.3
C8—O3—C17	118.0 (3)	C11—C10—C9	120.4 (3)
C12—O4—C14	104.9 (2)	C11—C10—C12	109.2 (3)
C14—O5—C15	116.3 (3)	C9—C10—C12	130.4 (3)
C5—N1—C4	106.2 (3)	C6—C11—C10	121.2 (3)
C5—N1—Pd1	112.1 (2)	C6—C11—Pd1	119.3 (2)
C4—N1—Pd1	141.7 (2)	C10—C11—Pd1	119.5 (2)
C12—N2—C13	107.0 (3)	N2—C12—O4	111.8 (3)
C12—N2—Pd1	112.0 (2)	N2—C12—C10	120.0 (3)
C13—N2—Pd1	141.0 (2)	O4—C12—C10	128.2 (3)
C2—C1—H1A	109.5	C14—C13—N2	106.6 (3)
C2—C1—H1B	109.5	C14—C13—H13A	126.7
H1A—C1—H1B	109.5	N2—C13—H13A	126.7
C2—C1—H1C	109.5	O5—C14—C13	137.7 (3)
H1A—C1—H1C	109.5	O5—C14—O4	112.5 (3)
H1B—C1—H1C	109.5	C13—C14—O4	109.7 (3)
O1—C2—C1	107.5 (4)	O5—C15—C16	107.2 (4)
O1—C2—H2A	110.2	O5—C15—H15A	110.3
C1—C2—H2A	110.2	C16—C15—H15A	110.3
O1—C2—H2B	110.2	O5—C15—H15B	110.3
C1—C2—H2B	110.2	C16—C15—H15B	110.3
H2A—C2—H2B	108.5	H15A—C15—H15B	108.5
O1—C3—C4	138.5 (3)	C15—C16—H16A	109.5
O1—C3—O2	111.2 (3)	C15—C16—H16B	109.5
C4—C3—O2	110.2 (3)	H16A—C16—H16B	109.5
C3—C4—N1	106.7 (3)	C15—C16—H16C	109.5
C3—C4—H4A	126.6	H16A—C16—H16C	109.5
N1—C4—H4A	126.6	H16B—C16—H16C	109.5
N1—C5—O2	112.5 (3)	O3—C17—H17A	109.5
N1—C5—C6	120.1 (3)	O3—C17—H17B	109.5
O2—C5—C6	127.3 (3)	H17A—C17—H17B	109.5
C11—C6—C7	119.9 (3)	O3—C17—H17C	109.5
C11—C6—C5	109.2 (3)	H17A—C17—H17C	109.5
C7—C6—C5	130.9 (3)	H17B—C17—H17C	109.5
C8—C7—C6	118.9 (3)		
			
C11—Pd1—N1—C5	−1.0 (2)	C17—O3—C8—C9	13.7 (5)
N2—Pd1—N1—C5	−1.9 (4)	C7—C8—C9—C10	0.8 (5)
Br1—Pd1—N1—C5	179.6 (2)	O3—C8—C9—C10	−179.9 (3)
C11—Pd1—N1—C4	178.5 (4)	C8—C9—C10—C11	1.1 (5)
N2—Pd1—N1—C4	177.5 (3)	C8—C9—C10—C12	179.5 (3)
Br1—Pd1—N1—C4	−1.0 (4)	C7—C6—C11—C10	0.7 (5)
C11—Pd1—N2—C12	−0.9 (2)	C5—C6—C11—C10	178.7 (3)
N1—Pd1—N2—C12	0.1 (4)	C7—C6—C11—Pd1	−178.6 (2)
Br1—Pd1—N2—C12	178.6 (2)	C5—C6—C11—Pd1	−0.6 (4)
C11—Pd1—N2—C13	179.9 (4)	C9—C10—C11—C6	−1.9 (5)
N1—Pd1—N2—C13	−179.2 (3)	C12—C10—C11—C6	179.4 (3)
Br1—Pd1—N2—C13	−0.6 (4)	C9—C10—C11—Pd1	177.5 (2)
C3—O1—C2—C1	−179.3 (4)	C12—C10—C11—Pd1	−1.3 (4)
C2—O1—C3—C4	−6.5 (7)	N2—Pd1—C11—C6	−179.4 (3)
C2—O1—C3—O2	173.7 (4)	N1—Pd1—C11—C6	0.9 (2)
C5—O2—C3—O1	179.6 (3)	N2—Pd1—C11—C10	1.2 (2)
C5—O2—C3—C4	−0.3 (4)	N1—Pd1—C11—C10	−178.5 (3)
O1—C3—C4—N1	179.9 (4)	C13—N2—C12—O4	−0.7 (4)
O2—C3—C4—N1	−0.2 (4)	Pd1—N2—C12—O4	179.8 (2)
C5—N1—C4—C3	0.6 (4)	C13—N2—C12—C10	180.0 (3)
Pd1—N1—C4—C3	−178.8 (3)	Pd1—N2—C12—C10	0.5 (4)
C4—N1—C5—O2	−0.9 (3)	C14—O4—C12—N2	0.8 (4)
Pd1—N1—C5—O2	178.8 (2)	C14—O4—C12—C10	−180.0 (3)
C4—N1—C5—C6	−178.7 (3)	C11—C10—C12—N2	0.5 (4)
Pd1—N1—C5—C6	1.0 (4)	C9—C10—C12—N2	−178.1 (3)
C3—O2—C5—N1	0.7 (4)	C11—C10—C12—O4	−178.8 (3)
C3—O2—C5—C6	178.3 (3)	C9—C10—C12—O4	2.7 (6)
N1—C5—C6—C11	−0.3 (4)	C12—N2—C13—C14	0.3 (4)
O2—C5—C6—C11	−177.7 (3)	Pd1—N2—C13—C14	179.6 (3)
N1—C5—C6—C7	177.4 (3)	C15—O5—C14—C13	−5.6 (7)
O2—C5—C6—C7	0.0 (6)	C15—O5—C14—O4	176.2 (3)
C11—C6—C7—C8	1.2 (5)	N2—C13—C14—O5	−178.1 (4)
C5—C6—C7—C8	−176.3 (3)	N2—C13—C14—O4	0.1 (4)
C6—C7—C8—O3	178.8 (3)	C12—O4—C14—O5	178.2 (3)
C6—C7—C8—C9	−2.0 (5)	C12—O4—C14—C13	−0.5 (4)
C17—O3—C8—C7	−167.0 (3)	C14—O5—C15—C16	−175.5 (4)

### Hydrogen-bond geometry (Å, º)

**Table:**

*D*—H···*A*	*D*—H	H···*A*	*D*···*A*	*D*—H···*A*
C2—H2*A*···Br1^i^	0.97	2.80	3.526 (5)	132
C16—H16*A*···O2^ii^	0.96	2.44	3.391 (5)	170

Symmetry codes: (i) −*x*+2, −*y*+1, −*z*; (ii) *x*−1, *y*, *z*+1.
